# EFL/ESL Teacher’s Resilience, Academic Buoyancy, Care, and Their Impact on Students’ Engagement: A Theoretical Review

**DOI:** 10.3389/fpsyg.2021.731859

**Published:** 2021-08-05

**Authors:** Mo Zhang

**Affiliations:** School of Music, Shandong Normal University, Jinan, China

**Keywords:** positive psychology, teacher resilience, academic buoyancy, care, student engagement

## Abstract

Teachers’ emotions and inner states play a crucial role in academia as they affect almost all aspects of their job. Language teaching as a stressful and tense profession is full of adversities and traumatic experiences, mandating teachers to be psychologically tough aside from their pedagogical readiness. In tune with this, the current study provides an overview of this area of research drawing on positive psychology and four fresh constructs, namely, resilience, buoyancy, care, and students’ engagement. More particularly, this review article presents the definitions, conceptualizations, dimensions, cognate terms, and influential factors related to each construct. Next, related empirical studies are reviewed to justify the results and position the current article in the body of knowledge in this domain. Finally, implications, gaps, and recommendations for future research are presented.

## Introduction

Teaching is a challenging and labor-intensive occupation due to its service-providing basis and accountability pressures (Mercer, 2020). That is why in language education which is full of challenges, teachers are considered as the precursor stakeholders (Nayernia and Babayan, 2019). In contrast to many other professions, second/foreign language teachers’ duty continues even after the class as they need to offer useful homework assignments, give feedbacks, and carry out assessments. In English as a foreign language (EFL) contexts, this pressure is multiplied because teachers usually face adversities caused by different sources, such as linguistic difficulties, (inter)cultural disparities, and instructional issues (King and Ng, 2018). This buttresses the claim that teachers are the “pillars” and “architects” of societies (Pishghadam et al., 2019, 2021). Therefore, knowing the teachers’ emotions and expectations is of utmost importance in academia. Aside from instruction and learning issues, teachers carry their own value systems, beliefs, mentalities, and behaviors to the class which can be indirectly inculcated in the learners (Haseli Songhori et al., 2018; Derakhshan et al., 2020). This justified the need for a paradigm shift from “learner psychology” to “teacher psychology” in which teachers’ emotions are the priority. To cause learning, teachers’ emotions, needs, and stressors must be identified and analyzed meticulously as the whole functionality of a teacher depends on the value and care given to him/her in an institution (Derakhshan et al., 2019).

Another critical issue in EFL contexts is the need for teachers to become resilient in the face of setbacks (Parsi, 2019). As pinpointed by Hong (2012), one of the best ways to minimize the job quitting rate of EFL teachers is raising their resiliency through appropriate strategies. The concept of resilience refers to one’s capability to recover and bounce back when he/she is encountered with adversities (Bernshausen and Cunningham, 2001). In other words, it is the ability to adapt with tough situations and improve one’s competence/skill when facing tensions and traumatic experiences (Bobek, 2002). It is a psychological construct in education which can considerably affect teachers and learners (Gu and Day, 2013). It is determined by many factors and influences teachers’ attitude, practices, commitment, sense of efficacy, identity, retention, satisfaction, and academic performance (Razmjoo and Ayoobiyan, 2019; Fathi and Saeedian, 2020). Teacher resilience is best illuminated in the *positive psychology* (PP) trend which highlights how people can thrive and have happier lives (MacIntyre et al., 2019). Instead of negative sides of teaching, PP urges the practitioners to focus on the power of positive emotions like joy, interest, passion, resilience, optimism, and the like to prevent the negative stressors. A resilient teacher, based on this conceptualization, is one who does not fringe when facing tough moments, responds positively to adverse experiences, has more satisfaction with his/her job, has more agency, has a sense of pride and accomplishment, and is competent (Howard and Johnson, 2004).

Furthermore, in an approval of the challenges inherent in ESL/EFL contexts, scholars have proposed a new cognate term for resilience called “academic buoyancy” which refers to the ability to manage the common challenges and adversities of educational life efficiently (Comerford et al., 2015). To put it simply, it is one’s capability to endure and overcome the challenges, stressors, and setbacks in his/her instructional career (Verrier et al., 2018). Buoyancy, like other constructs, is affected by both internal and external factors. Internal factors, such as self-efficacy, self-confidence, motivation, and agency, can influence one’s academic buoyancy. Conversely, socio-cultural contexts, educational milieus, and stakeholders as external forces can also determine one’s academic buoyancy (Comerford et al., 2015). Like resilience, the concept of academic buoyancy has its roots in PP and takes a proactive approach to cope with challenges and adversities in academia. Rather than focusing on negative sides and stressors, academic buoyancy considers one’s emotions, capacities, and strengths to increase his/her wellbeing and psychological development (Jahedizadeh et al., 2019). In other words, it can be asserted that academic buoyancy is a positive form of resilience that highlights one’s degree of endurance in the face of minor academic challenges. Academic buoyancy has been identified to affect various aspects of the teaching-learning cycle, like students’ achievement, motivation, self-efficacy, self-esteem, classroom enjoyment, self-regulation strategies, lowering stress and anxiety, increasing class participation, test performance, and so forth (Putwain et al., 2015; Yun et al., 2018).

These objectives are obtained only by a friendly and positive rapport between the teacher and students in the classroom. In a context full of adversities, teacher-students’ supportive relationships can function as a panacea and a driving force for improving students’ achievement and teachers’ performance (Irajzad and Shahriari, 2017). When teachers care about their pupils, they provide a reason for the students to invest their time in learning a subject. The whole education is about caring and showing the students that they are valued and considered (Ware, 2006). However, in reality, many students lose their motivation to learn as they feel that they are completely forgotten by their teachers. This sense of alienation damages teacher-students’ rapport and leads to a care crisis in academia (Schat, 2018). As research shows, teachers’ care provides different positive outcomes, such as higher student attendance, increased effort, higher achievement, higher motivation to learn, higher autonomy and agency, lower anxiety, and lower dropout rate (Bieg et al., 2013; Ma et al., 2018). Moreover, it is essential to note that the three constructs of teacher’s resilience, buoyancy, and care largely affect students’ engagement as well. Students’ engagement refers to their degree of involvement in the class during instructional activities which may continue for minutes (Skinner and Pitzer, 2012). Engagement is an explicit indicator of motivation and motivation theories which can considerably affect each other (Elliott and Tudge, 2012; Reeve, 2012). It is a dynamic construct upon which numerous internal and external factors exert influences (Guilloteaux, 2016). Students’ engagement has a significant role in language teaching and learning since the students who are engaged are usually energized, dedicated, attracted, effortful, and determined in the learning process (Abas, 2015; Guilloteaux, 2016).

The concept of engagement has different dimensions, including behavioral, emotional (affective), cognitive, agentic, academic, and social engagement (Reschly and Christenson, 2012; Oga-Baldwin, 2019). All of these components can be improved by teachers’ positive instructional practices (McKellar et al., 2020). Research indicates that students’ engagement strongly predicts different academic outcomes and is improvable through training (Eccles, 2016). More specifically, it has been identified to correlate with learners’ achievement, psychosocial adjustment, effective learning, and success (Chase et al., 2015; Jang et al., 2016). Moreover, as pinpointed by Quin (2017), the degree and quality of teacher-student rapport and the overall classroom climate directly influence students’ engagement. Thus, EFL teachers’ caring behaviors and being academically resilient and buoyant play a crucial role in creating engagement among their students.^1^ When EFL teachers are tough in the face of adversities inherent in L2 education and show positive regards toward their students’ emotions and desires, the whole process of education succeeds. Motivated by this, the present review article aimed to go through studies conducted in this line of research, show the existing gaps, and offer future directions to avid researchers in EFL/ESL contexts.

## Theoretical Background

### Positive Psychology and the Broaden-and-Build Theory

Undoubtedly, emotions play an important role in foreign language learning and teaching (Dewaele et al., 2019). As the dominance of cognitive perspectives reduced, the emotional aspects of L2 education came into vogue among scholars (Prior, 2019). Nowadays, teachers’ and students’ affects are the central parts of many research studies with the emergence of positive psychology (PP). In essence, PP tries to demystify how people flourish (Mercer and MacIntyre, 2014) by highlighting the power of positive sides of life (Seligman, 2006). Moreover, this school of psychology cares for people’s positive emotions, makes attempts to raise their passion and engagement, and provides a meaning for their life (Seligman, 2006).

Researchers following PP, run studies on variables, like interest, courage, enjoyment, perseverance, clarity, wellbeing, creativity, credibility, flow, care, optimism, happiness, hope, resilience and the like. PP rests on three main pillars of *positive subjective experience* (emotions), *positive individual traits* (individual characteristics), and *positive institutions* (contexts) (Mercer and MacIntyre, 2014; Seligman and Csikszentmihalyi, 2014). Positioning itself in applied linguistics, PP has become the focus of numerous studies on positive affect and emotions in learning in different educational contexts (Dewaele, 2015). The constructs of this school are useful for L2 learners and teachers especially for enlightening their resilience, improving their motivation and engagement, and diminishing the impact of negative emotions (Dewaele et al., 2019).

One of the by-products of PP which is pertinent to this article is the Broaden-and-Build Theory developed by Fredrickson (2004). The theory draws a demarcation between positive and negative emotions, but emphasizes more on the role of positive emotions in human’s flourishing and success. According to Mercer and MacIntyre, (2014), positive emotions have five important functions: (1) They broaden thought-action repertoires, (2) minimize the effects of negative emotions, (3) develop psychological resiliency, (4) provide personal resources, and (5) produce wellbeing. Conversely, negative emotions have harmful impacts on people as they compress one’s thought-action repertoires and weaken his/her performance. Moreover, this theory underscores individuals’ internal ability to use positive emotions (Doney, 2013) and offers a clarification for what a resilient behavior means from a psychological viewpoint (Rizqi, 2017). Other than the power of positive emotions, this theory focuses on one’s ability to build a block that defends him/her from a negative effect. More importantly, this theory considers positive emotions as the primary fuels of creating a resilient person and the cause of ideal functioning (Fredrickson, 2004). In L2 education, positive emotions are significant in that an academic context which values stakeholders’ affective factors and stimulates students’ determination for chasing of their thoughts and actions upsurges motivation, engagement, and learning (Rahimi and Bigdeli, 2014). Likewise, teachers can utilize positive emotions to wipe out negative emotions and convert them into positive ones (Fredrickson, 2004). Hence, positive emotions of all stakeholders are very important in EFL/ESL contexts as they are the prerequisites and predictors of many other variables and constructs of L2 education.

### The Concept of Resilience

The construct of resilience has emanated from psychiatry and developmental psychology (Hiver, 2018) and has been defined differently by researchers. The conceptualizations are generally divided into two perspectives: One considers resilience as an individual characteristic which is revealed during hard times, while the other regards it as a concept beyond intrapersonal traits to encompass socio-cultural factors as well. In simple terms, resiliency is the ability of a person to manage challenges and “bouncing back” in the face of adversities (Bernshausen and Cunningham, 2001; Beltman et al., 2011). Moreover, resilience has been seen as a process of positive adaptation which can improve through promoting some specific competencies.^2^

The reasons behind multiple definitions for resilience are disagreements over its locus (internal or external to the person), nature (process or product), operationalization, and whether it is a fixed attribute or a dynamic one (Cohen et al., 2011; Gu and Day, 2013). Now, resilience is conceptualized as a personal attribute which is both intrapersonal and interpersonal and emerges dynamically from an interplay of different factors (Ungar, 2012). Similarly, Gu (2018) considers resilience as a “context- and role-specific” concept which goes beyond the maintenance of balance, being committed, and has agency.

From Cohen et al.’s (2011) viewpoint, resilience is a dynamic process dependent on many psychological, biological, and environmental-contextual processes besides individual traits, familial aspects, and the social milieu in which one lives. Similarly, Kostoulas and Lämmerer (2018) regarded resilience as an attribute emerging from a resilient system and comprises three groups of correlating constructs, that is, inner strengths, external support structures, and learned strategies. Despite these various definitions for resilience, there are two commonalities and conditions among all of them. They include the presence of an adversity and a positive adaptation to it (Castro et al., 2010). In teaching profession, resilience is a vital key to understand both teaching and learning processes (Hui and Abdullah, 2020) and happens when individuals connect their personal and contextual resources and employ effective strategies to overcome difficulties and sustain their wellbeing (Greenier et al., 2021; Mansfield et al., 2016).

### Common Features of Resiliency

In the available literature, different characteristics have been identified for resiliency. According to Tait (2008), resilient teachers usually have high job satisfaction levels, respond positively in tense circumstances, exhibit effective strategies for managing difficult situations, and are highly efficacious and emotionally intelligent teachers. Moreover, Howard and Johnson (2004) concluded that resilient teachers have a feeling of pride and fulfillment, own behavioral management skills, can limit negative emotions, empathize with their students, exhibit a sense of agency, have moral purposes, and are competent and supportive. Additionally, Taylor (2013) proposed that teachers with high resiliency have an elastic locus of control, autonomy, optimism, commitment, positive rapports, and enjoy educational changes.

Likewise, Khanshan and Yousefi (2020) argued that resilient teachers are self-confident, optimistic, able to develop close relationship with others, motivated, competent, teacher-researchers, and attentive to critical incidents. Additionally, Day and Gu (2014) argued that resilient teachers have long lasting effectiveness and commitment.

### Factors Affecting Teacher Resilience

As a meta-construct, teacher resilience is affected by a range of individual, social, familial, organizational, and contextual factors called *protective factors* and *risk factors* which aid people fight back tensions (Stavraki and Karagianni, 2020). These factors come in environmental (contextual) and internal (individual) forms. Concerning the individual protective factors, research indicates that inner drives, like intrinsic motivation, altruistic motive, and self-efficacy, are the main personal resources that teachers can employ to resist challenges (Mansfield et al., 2016). As for individual risk factors, low self-esteem, negative self-beliefs, lack of motivation, lack of confidence, weak relationship with others, and mismatch between personal beliefs and actual practices are the most common factors (Jenkins et al., 2009). In addition to these, positive attitude (Stavraki and Karagianni, 2020), positive emotions (Mercer et al., 2016), enthusiasm (Yost, 2006), persistence (Le Cornu, 2009), perseverance (Gu and Day, 2007), empathy (Jennings et al., 2011), and hope (Huisman et al., 2010) can affect teachers’ resilience, too.

Concerning the contextual protective factors, scholars argue that a robust, caring, open, and efficient leadership style and rapport with peers and colleagues are the most prevalent factors (Karagianni and Papaefthymiou-Lytra, 2018). Considering contextual risk factors, it has been purported that ineffective class management, unsupportive leadership, dearth of resources, and weak rapport are the most obstinate sources which endanger teachers’ resilience (Gibbs and Miller, 2014; Stavraki and Karagianni, 2020). Aside from these factors, academic degree, income, demographic background, and occupational characteristics may also affect teachers’ resilience.

## The Notion of Academic Buoyancy

Academic buoyancy has stemmed from PP which has put its emphasis on the role of emotions in education (Agudo, 2018). In academic contexts which are full of adversities, buoyancy refers to an individual’s ability to navigate and cope with the difficulties that occur (Martin and Marsh, 2019). It is a psychological construct which mirrors the routine academic setbacks in a positive context (Jahedizadeh et al., 2019). In L2 education, buoyancy beckons to the ability to negotiate and overcome the ups and downs of language learning and teaching (Yun et al., 2018). This construct can be affected by several factors internal and external to the individual. Internal factors include personality traits, like autonomy, motivation, self-efficacy, confidence, and self-esteem (Anderson et al., 2020). Oppositely, external factors refer to the contextual factors available in the educational environments which are crucial in shaping and developing interpersonal communication skills and academic buoyancy (Comerford et al., 2015).

There are some fundamental principles behind academic buoyancy, including (1) drawing on strengths rather than weaknesses, (2) taking proactive instead of reactive approaches to adversities and challenges, and (3) considering the “many” and the “healthy” cases rather than extreme ones in making propositions (Martin and Marsh, 2019). That is why, academic buoyancy is said to adapt the PP orientation and is the positive version of resilience.

### The Cognates of Academic Buoyancy

The construct of academic buoyancy has been associated with some cognate terms, such as resilience, immunity, hardiness, and *coping*, all of which have their own specific denotations. Saying this, there must be drawn a demarcation line among these terms. One of these cognates is *resilience* which seems equal to buoyancy, but in reality, it differs from it. While both concepts have a similar theoretical ground, resilience has limited applicability in academia as it does not clarify the adversities that routinely happen in one’s academic life (Phan and Ngu, 2014). Moreover, resilience has definitional, sampling and population, operational, and methodological distinctions with academic buoyancy (Jahedizadeh et al., 2019). Furthermore, it can be argued that resilience focuses on the adversities of a small and extreme group of cases, while buoyancy considers “many and healthy” individuals’ typically arisen experiences of challenge in academia (Martin and Marsh, 2019).

Another similar concept is *immunity* which refers to the armoring and defensive mechanisms that are used to minimize the challenges, disturbances, and damages imposed on one’s motivation, identity, and practice (Hiver, 2017). It differs from resilience and buoyancy in that it is spontaneous, double-edged (i.e., productive and counterproductive), and integrated into one’s professional identity (Hiver, 2017). Another synonymous term is *hardiness* which is claimed to be a personality trait that is able to fight and minimize the impacts of stress on one’s performance (Hiver and Dörnyei, 2017). Lastly, *coping* is also a related notion here which refers to the strategies and techniques that are utilized to either cure the stressors or alter how they are perceived by the person (Somerfield and McCrae, 2000). It is worth noting that in many cases the boundaries between these cognates are not clear and there are many overlaps among them which need further elucidations in future studies.

## The Conceptualization of Care in Education

In education, teacher-student rapport is one of the most important factors which causes many positive outcomes, such as developing interpersonal communication skills, intelligence, motivation, achievement, and confidence (Irajzad and Shahriari, 2017; Derakhshan et al., 2019). The concept of teacher care was first proposed by Rogers and Webb (1991) who described care as teachers’ behaviors and practices that foster the establishment of a strong, positive, and harmonious interpersonal relationship with their students. It involves forming a classroom atmosphere wherein both the students and the teacher simultaneously feel respected (Ware, 2006). Care also refers to an encouraging, trusting, and supportive relationship between teacher and students which can produce different positive outcomes, such as willingness to learn, having a sense of relatedness, higher attendance, increased learning investment, improved achievement, higher motivation, and lower anxiety (Bieg et al., 2013; Derakhshan et al., 2019).

Considering teacher care as a relational practice, Noddings (1992) introduced six domains for the concept of care, including *caring for oneself*, *caring for close others, caring for faraway others*, *caring for non-individual life*, *caring for things*, and *caring for beliefs*. Out of these, caring for oneself and caring for others are the most significant ones in educational arena (Derakhshan et al., 2019; Pishghadam et al., 2021). Moreover, Noddings (1992) maintained that caring relationships have three characteristics, namely, *engrossment* (accepting students’ feelings and experiences), *commitment* (equality of care), and *motivational shift* (shifting the focus from self to students as others). Despite its significance, care is still unclear and vague for many teachers who do not know what the indicators and ethics of a caring relationship are. Most of the time, teachers assume that they care for their students, while in reality a crisis and loss of care happen in many educational contexts (Schat, 2018). To tackle these, the understanding of care in education should permeate into teachers’ pedagogical content knowledge and useful professional development courses are required for teachers to realize that care is not something assumed but a teachable construct.

## Student Engagement: The Definitions and Dimensions

Student engagement is one of the most critical issues in all educational systems which generates energy, investment, and success in academic contexts (Phillips, 2015). It is a buzzword which has caught the attention of many scholars over the past couple of decades due to its positive outcomes in L2 education (Eccles, 2016). As defined by Skinner and Pitzer (2012), engagement concerns the quality of students’ involvement in classroom activities which may last for a while. It is an overt sign of intrinsic motivation and the outcome of an interplay of different individual and contextual factors (Elliott and Tudge, 2012).

Owing to its significance, engagement has been conceptualized differently by different scholars, yet the most common conceptualization is that the concept is a multi-dimensional and a meta-construct which involves a range of dimensions. The dimensions are behavioral, emotional (or affective), cognitive, agentic, academic, and social (Reschly and Christenson, 2012). *Behavioral engagement* concerns students’ compliance and active involvement in the activities (e.g., paying attention, participation, listening, task involvement, asking questions, and doing the homework), while *Emotional engagement* refers to students’ internal states and their affective reactions in the learning process (e.g., students’ interest, enjoyment, having fun, happiness, boredom, and anxiety). Besides, *cognitive engagement* concerns students’ psychological investment in learning and using complex learning strategies during an activity. The next dimension is *agentic engagement* which is associated with students’ contribution to the enhancement of learning and teaching quality. Similarly, a*cademic engagement* concerns a student’s psychological and behavioral efforts and investment in learning and mastering knowledge and skills of an academic work (Fredricks et al., 2004). The final dimension here is *social engagement* which is related to students’ involvement in a variety of tasks which are intended to stimulate social interaction and problem-solving in students.

### Factors Affecting Students’ Engagement

**Figure 1:**
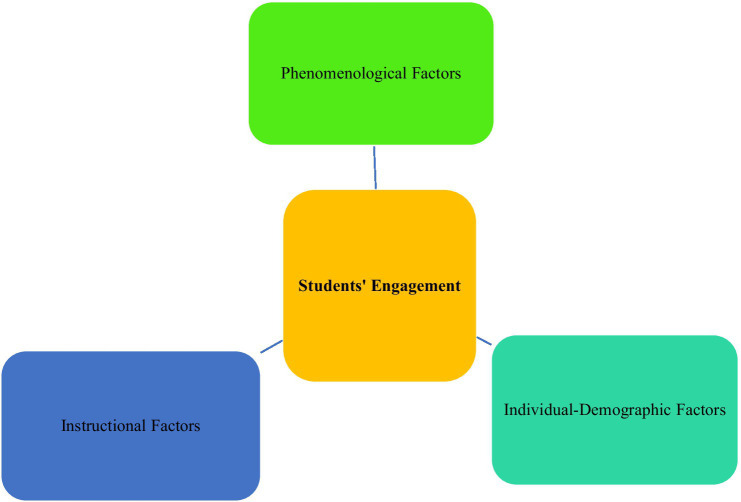
Factors influencing students’ engagement.

As stated earlier, engagement is a dynamic and multi-faceted characteristic which can be affected by various factors (Collins, 2014). In a landmark study, Guilloteaux (2016) classified the influencing factors into (1) *phenomenological factors*, such as task difficulty, ability level, culture, task type, and task value, (2) *individual-demographic factors*, including age, gender, and academic grade, and (3) *instructional factors*, such as teachers’ actions, behaviors, motivation to teach, ability, and instructional style (Figure 1). As engagement is malleable, there are also other factors and variables which can affect it, including different personality traits, time, motivation type, self-esteem, self-efficacy, confidence, interest, and the like. In sum, students’ engagement as one of the most important issues in academia which can predict many academic outcomes needs sufficient attention among practitioners. They should utilize various tasks and techniques in order to minimize the impact of intervening variables and at the same time reinforce facilitating factors in this regard.

## Empirical Studies

Perusing the literature on teachers’ resilience, buoyancy, and care and their impacts on students’ engagement, one can easily realize that ample empirical studies have been conducted though buoyancy and care need further explorations. Considering teachers’ resilience, many studies have approved its predictive power and positive outcomes, such as improved attitude, effective practices, higher commitment, higher level of efficacy, promoted identity, high retention, satisfaction, and academic performance. Additionally, Stavraki and Karagianni (2020) conducted a quantitative study on Greece EFL teachers’ resilience in light of their demographic, occupational, and school/class characteristics. Their results indicated that such variables did not affect the participants’ resilience level. Likewise, Rizqi (2017) ran a case study in Indonesia on the role of stress in shaping teachers’ resilience and found that teachers’ positive emotions, supportive relationships, and calm working environment reduce stress and help changing it to resilience. Similarly, Entesari et al. (2020) conducted a mixed-methods study on novice and experienced EFL teachers’ resiliency and identified that experienced teachers are more resilient and employ more coping strategies when they face adversities. Moreover, in China, Qiong et al., 2019 conducted a correlational study on work conditions and relational trust and teachers’ resilience and found that the quality of these variables can significantly predict teachers’ resilience.

Likewise, Shirazizadeh et al. (2019) examined the relationship among role stress, reflection, and resilience using questionnaires and an interview. The results revealed a significant positive relationship between reflection and resilience, while reflection and role stressors had a negative correlation. Trying to see the predictive power of resilience and creativity, Parsi (2019) collected quantitative data from 122 EFL teachers and identified that resilience significantly predicted their creativity.

As for academic buoyancy, research endorsed its positive impacts on learners’ improved academic achievement, motivation, self-efficacy, self-esteem, classroom enjoyment, self-regulation strategies, class participation, test performance, and lowering their stress and anxiety (Putwain et al., 2015; Yun et al., 2018). Moreover, Martin and Marsh (2019) investigated the relationship between buoyancy and adversity and if academic buoyancy protects students in the face of adversity. The results indicated that academic buoyancy significantly predicted the participants’ subsequent academic adversity. Furthermore, Jahedizadeh et al. (2019) explored buoyancy at higher education level in Iran and developed a scale to assess buoyancy in relation to GPA, gender, and educational level. In the end, they found that buoyancy has correlations with those factors and is able to improve sustainability among EFL students. Despite its significance, the concept of academic buoyancy has not received due attention from scholars in EFL/ESL contexts.

Care is another recent construct which has seen a burst of interest from scholars who highlight the criticality of a caring relationship between the teacher and the students. There is sufficient evidence that teachers’ care has a strong and positive relationship with student attendance, effort, achievement, motivation to learn, autonomy and agency, lower anxiety, and lower dropout ratio (Bieg et al., 2013; Pishghadam et al., 2015). Furthermore, Derakhshan et al. (2019) conducted a study on EFL teachers’ conception of intelligence, care, feedback, and stroke in Iran. They gathered their data from 200 EFL students and 30 EFL teachers through four scales but did not find a significant relationship between teachers’ conception of intelligence and care. Nevertheless, they identified a significant relationship between teachers’ conception of intelligence, feedback, and students’ stroke. Likewise, Kordi et al. (2012) examined the effect of teachers’ care on EFL students’ writing and found that students’ macro-level structures of writing had improved as a result of a caring relationship with their teacher. Additionally, Lavy and Naama-Ghanayim (2020) ran a multi-level study on the association and predictive power of 33 teachers on 675 students’ self-esteem, wellbeing, and school engagement. The results demonstrated that teachers’ caring rapport mediates the relationship among these constructs. Moreover, the students attributed this impact to teachers’ perceived meaning of their profession.

As the last variable, engagement refers to the quantity and quality of students’ involvement in classroom activities (Hiver et al., 2021). It is one of the fundamental cores of language learning which affects students’ interest, motivation, self-efficacy, and persistence (Egbert, 2020). Similarly, Dincer et al. (2019) scrutinized the antecedents and outcomes of 412 Turkish EFL students’ classroom engagement in a mixed-method research and found that teachers’ autonomy support in the classroom predicted students’ need satisfaction and self-determined engagement. Correspondingly, students’ engagement predicted academic achievement and absenteeism in English language classes. Likewise, Guilloteaux (2016) explored Korean EFL students’ engagement and found that 87% of the students’ engagement was away from their optimal achievement. More specifically, 50% of the students were disengaged and 37% were moderately engaged in EFL classrooms. Similarly, Tian et al. (2020) examined 1,428 students’ engagement level in Chinese international education and identified that their engagement is less than satisfactory levels. Although research on engagement abounds, most of them are theoretical and quantitative (Hiver et al., 2021) and empirical studies are scanty to be reviewed here. However, there are some recommendations for future research on this construct in the following section.

## Implications and Future Directions

In this article, it was pinpointed that teaching is a labor-intensive profession which places stress, tension, and accountability on teachers. That is why in many contexts teachers may feel demolished by such unbearable pressures and leave the job. Admitting these, now many educational systems widely embrace the significance of teachers’ emotions and inner states after the emergence of positive psychology. To perform better, teachers should be resilient and academically buoyant in the face of setbacks, like cultural mismatches, linguistic disparities, and language identity (re)formations during instruction. Moreover, it was concluded that teachers’ resiliency and buoyancy depend on several internal and external factors. To survive in such a tense context, teacher should establish a caring rapport with their pupils and engage them in the class through appropriate strategies.

In this study, the researchers drew on PP and presented the theoretical foundations beneath four important constructs of teacher’s resilience, academic buoyancy, care, and student’s engagement. More specifically, their definitions, dimensions, influencing factors, and empirical studies were provided concisely. According to this review article, this domain has valuable implications for different stakeholders, including students, teachers, teacher-trainers, materials developers, and L2 researchers. Considering EFL students, this study is beneficial in that it can increase their knowledge of the joint nature of education. That is to say, students are no longer regarded as passive members of the instruction but active agents who can help their teachers by forming a positive rapport and a caring environment. Students can also reduce the challenges imposed on their teachers and, in turn, their motivation, engagement, achievement, and resiliency improve as well. Regarding EFL teachers, the results are precious in that they improve teachers’ awareness of the inherent difficulties in their job and how their determination, positive emotions, and resiliency can affect different areas of students’ personality and learning. They can use effective coping strategies to fight the traumatic events in teaching and convert the difficulties into opportunities for themselves and their students. Moreover, the results can show them the significance of establishing a caring relationship in the class to improve students’ learning, motivation, self-efficacy, interest, engagement, and the like.

Also, teacher trainers can enjoy the propositions made in this article by offering training courses, seminars, workshops, and professional development programs in which the importance of teachers’ positive emotions and patience and toughness in the face of challenges of the profession is taught to pre-service and in-service EFL teachers. They can also offer useful techniques in TTC classes through which resiliency, buoyancy, and care become an explicit part of teachers’ pedagogical content knowledge rather than something assumed in teachers. Additionally, materials developers can use the findings of this study and write materials in which teachers’ emotions are mirrored in the activities. Likewise, they can design activities which can boost teacher-students relationship which, in turn, improves students’ engagement and attainment. When the textbook echoes teachers’ concerns, their stress reduces and a defensive mechanism is constructed to help them tackle the challenges of teaching a foreign language. Finally, L2 researchers can profit from this study in that they can conduct more studies on other related constructs in this research zone.

Although research on this area has brought about insightful ideas, there are still many backdrops and unexplored avenues left to future avid investigators. One of such shortcomings is that most of the conducted studies on teacher’s resilience, buoyancy, care, and student’s engagement are quantitative and based on self-reported, one-shot, and retrospective data instead of real-time and introspective data. As such variables are intra-psychic factors which alter through time, it is wise to scrutinize them qualitatively in case and longitudinal studies. Furthermore, future studies can be ran using diary, portfolio, observation, and reflective journals to see how these constructs are made and affect teachers and students in real time circumstances. Moreover, examining this line of research in ESP and EAP contexts is rare. Therefore, studies can be done on how these constructs work in these contexts even by comparing them with their process in EFL settings. Another flaw is that most of the studies in this area have used non-random sampling which limits their generalizability; hence, researchers are suggested to use random sampling in experimental studies to examine the influence of treatment on these constructs.

The role of culture and socio-cultural context in the (re)construction of the variables of interest in this study has not caught due attention. So, cross-cultural studies can be conducted in different cultural settings to focus on the trajectories and social indicators of such constructs (see Pishghadam et al., 2021). Another limitation is the dominance of teachers’ perspectives in this area, while these constructs can be examined from the viewpoints of other stakeholders, too. Among the variables, teachers’ buoyancy and care have been limitedly studied in EFL/ESL contexts in comparison with resilience and engagement. Consequently, researchers can investigate these two variables in relation to other teacher-related variables, like self-belief, self-efficacy, motivation, commitment, confidence, wellbeing, professional identity, immunity, and self-concept. The mediating role of some demographic and occupational factors in this strand has also gained scant attention. For instance, the impact of teachers’ teaching experience, educational background, and socio-economic status can be examined by future researchers. These flaws show that this line of research is still nascent in EFL/ESL and needs more research.

## Author Contributions

MZ has made a direct and intellectual contribution to the work and got it ready for its publication.

## Conflict of Interest

The author declares that the research was conducted in the absence of any commercial or financial relationships that could be construed as a potential conflict of interest.

## Publisher’s Note

All claims expressed in this article are solely those of the authors and do not necessarily represent those of their affiliated organizations, or those of the publisher, the editors and the reviewers. Any product that may be evaluated in this article, or claim that may be made by its manufacturer, is not guaranteed or endorsed by the publisher.

## Footnotes

^1^DeVito, M. (2016). Factors influencing student engagement. unpublished certificate of advanced study thesis. Fairfield, CT: Sacred Heart University.

^2^Eldridge, M. (2013). Understanding the factors that build teacher resilience. unpublished doctoral dissertation. University of London.
